# Assessment of *Trichogramma japonicum* and *T. chilonis* as Potential Biological Control Agents of Yellow Stem Borer in Rice

**DOI:** 10.3390/insects8010019

**Published:** 2017-02-08

**Authors:** Rui Tang, Dirk Babendreier, Feng Zhang, Min Kang, Kai Song, Mao-Lin Hou

**Affiliations:** 1 MoA-CABI Joint Laboratory for Bio-Safety, Institute of Plant Protection, Chinese Academy of Agricultural Sciences, 2 West Yuan-Ming-Yuan Road, Haidian District, Beijing 100193, China; r.tang@cabi.org (R.T.); f.zhang@cabi.org (F.Z.); 2 CABI Europe-Switzerland, Rue des Grillons 1, Delémont CH-2800, Switzerland; 3 Plant Protection and Quarantine Station, Dehong Prefecture Agriculture Bureau, Dehong 678400, China; kangmin318@sina.com; 4 Dryland Farming Institute, Hebei Academy of Agricultural and Forestry Sciences, Hengshui 053000, China; hs_kai@126.com; 5 State Key Laboratory for Biology of Plant Disease and Insect Pests, Institute of Plant Protection, Chinese Academy of Agricultural Sciences, Beijing 100193, China

**Keywords:** *Trichogramma*, yellow stem borer, parasitism rate, field release

## Abstract

Two species of *Trichogramma* wasps were assessed for their effectiveness against yellow stem borer *Scirpophaga incertulas*. A laboratory cage test with *T. japonicum* and *T. chilonis* showed that both species parasitized yellow stem borer egg masses at 60.0% ± 9.13% and 40.7% ± 7.11%, respectively, with egg parasitism rates of 15.8% ± 22.2% for *T. japonicum* and 2.8% ± 5.0% for *T. chilonis*. Once the host eggs were parasitized, emergence rates were high for both species (95.7% ± 0.12% for *T. japonicum* and 100% for *T. chilonis*). In paddy field trials, the two *Trichogramma* species were released at three densities (50,000/ha, 100,000/ha and 200,000/ha) in Southwestern China. Egg mass parasitism was 9% ± 7.7% for *T. japonicum* and 15% ± 14.1% for *T. chilonis*, and again only a relatively small fraction of eggs was successfully parasitized. No clear conclusion could be drawn on the most efficient release rate as no significant differences were found among the three release rates. A comparison of field-collected *T. japonicum* with *T. japonicum* and *T. chilonis* mass reared on *Corcyra cephalonica* showed significantly larger body size and ovipositor length in field-collected wasps, suggesting potentially higher effectiveness on yellow stem borer eggs after at least one generation on the target host. Factors contributing to the low field parasitism rates are discussed.

## 1. Introduction

Rice (*Oryza sativa* L.) is the most important crop in the world [1] and plays a central part in Asian food security [2]. This crop is widely distributed especially in Southern parts of China [3] where rice accounts for 88.7% of the total agricultural acreage [3]. Yunnan Province is located in the Southwest of China, and is considered to be part of the Greater Mekong Subregion, together with Myanmar, Laos, Cambodia, Vietnam and Thailand [4]. Rice covers over half of the cropping lands in this region [5] and is considered to be the most important crop there [5,6]. However, rice production in this area is suffering from serious pest and disease damage [7,8,9,10] causing substantial yield losses every year [6,11,12,13].

Key pests of rice include striped stem borer (*Chilo suppressalis* Walker, Crambidae) [6], yellow stem borer (YSB) (*Scirpophaga incertulas* Walker, Crambidae) [13], pink borer (*Sesamia inferens* Walker, Noctuidae) [14], rice leaf folder (*Cnaphalocrocis medinalis* Guenée, Crambidae) [13], rice plant hopper (*Nilaparvata lugens* Stål, Delphacidae) [15] and rice green semilooper (*Naranga aenescens* Moore, Noctuidae) [16]. Among those, the yellow stem borer is considered to be the most important pest of rain-fed low land and flood-prone rice ecosystems [17]. Populations of this pest substantially increased within one decade recently in paddy fields in Yunnan Province along with continuous promotion of double cropped rice in this area [18].

Conventional control methods for Lepidoptera pests in paddy fields usually involve the application of agrochemicals [5]. However, these methods may cause damage to the environment and lead to food safety issues, particularly because broad-spectrum insecticides of considerable toxicity are generally used [19]. Optimized methods with less environmental impact and high sustainability are in demand, such as releasing biological control agents [11]. So far, many studies have demonstrated that *Trichogramma* wasps can successfully control Lepidopteran pests by parasitizing their eggs [6,11,20,21,22]. Major success stories have been reported from maize crops and *Trichogramma* has been introduced in many maize growing areas worldwide [20]. In rice, *Trichogramma* has been studied for management of key Lepidopteran pests, however, despite promising results, *Trichogramma* is not yet used commercially in paddy fields. Recent findings from China indicate that *Trichogramma* releases may be considered practical for control of striped stem borer and rice leaf folder [6,21,22]. However, it is less clear whether the yellow stem borer can also be controlled by *Trichogramma* wasps as less work has been done on this species so far [23]. Furthermore, yellow stem borer egg masses consist of several layers of eggs and are protected by a cover of hairs [17]. These protection mechanisms could lead to difficulties for *Trichogramma* females to reach the eggs, especially the lower egg layers; a problem potentially linked to body size and ovipositor length of the wasps. From field surveys conducted in Indian rice fields, there are hints indicating that yellow stem borer eggs may not be effectively parasitized under natural conditions [24,25]. On the other hand, more positive results have been reported from a field survey in China showing rather high parasitism rates of yellow stem borer eggs in the range of 46.7% to 79.1% [26,27]. In general, there have been very few attempts to control yellow stem borers by inundative releases of *Trichogramma*, and none have occurred in China. However, one such experiment achieved a yield increase of 12% at maximum compared to the untreated control in paddy fields when releasing 200,000 *T. japonicum* adults/ha in two or four split applications [28]. Our aim was to assess the performance of two different *Trichogramma* species in both field cage and field release tests in order to explore their potential as biological control agents of yellow stem borer.

## 2. Materials and Methods

### 2.1. Insects and Plants

*T. chilonis* (Ishii) was collected from striped stem borer eggs in Cuijia Township (25°55′46″ N, 109°37′51″ E), Xing’an County, Guangxi Province, China during June 2011, and *T. japonicum* Ashmead was collected from yellow stem borer eggs in Husa Township (24°27′48″ N, 97°53′24″ E), Dehong Prefecture, Yunnan Province, China during July 2012. Both species were reared at Tianyi Biological Control Company Inc., Hengshui, Hebei Province, China, on *Sitotroga cerealella* Olivier with regular rejuvenation provided (host eggs were altered every 15 generations between *Sitotroga* and *Corcyra cephalonica* Stainton and backcrossing was conducted annually using field-collected wasps) (Appendix Table A1). For field experiments conducted in July and August of 2013 in Husa Township, tricho-cards with approximately 500 wasps emerging per card were used. The tricho-cards are of a folded design for protection from rain, sunshine, and natural enemies, with parasitized substitute host eggs glued on the inner side and a hole on one end for hanging on rice leaves. The tricho-cards were delivered several times to the experiment site so as to coincide with the field release timing.

Yellow stem borer moths were collected from nearby rice fields by sweep nets and then caged for oviposition on rice plants. Egg masses were collected daily from the plants and used within 24 h in the tests. Rice plants at heading stage randomly selected from the paddy fields near the experimental site were transplanted together with soil to pots and used for YSB oviposition and in cage tests.

### 2.2. Performance of the Trichogramma Species on Substitute Host

A test was conducted to measure the performance of both wasp species on the substitute host *S. cerealella*. Five hundred fresh, UV-sterilized *Sitotroga* eggs were each exposed to 100 *T. japonicum* and *T. chilonis* wasps in a Petri dish. After 48 h, the wasps were removed and the eggs were transferred to a Petri dish lined with moistened filter paper under room temperature. The emerged *Trichogramma* adults were counted and sexed daily. The measurement was replicated three times for each species.

### 2.3. Cage Tests for Parasitism

Cage tests were conducted to assess the capacity of *T. japonicum* and *T. chilonis* to parasitize YSB eggs. Rice hills at early heading stage were collected from the field and grown in pots in cylindrical cages (120 cm in height and 35 cm in diameter) made of wire and fine netting at natural conditions (1 to 2 hills per cage). Twenty pairs of yellow stem borer adults were introduced into a cage for oviposition. After 24 h, 1/5 of a tricho-card of either *T. japonicum* or *T. chilonis* that was ready to emerge was introduced per cage, corresponding to an estimated 100 parasitized eggs for each of the two species tested. Four replicates (cages) were set up for each *Trichogramma* species. Forty-eight hours after introduction of tricho-cards, all YSB egg masses were cut down together with rice leaves and placed individually on a moistened filter paper in glass tubes (15 cm in length and 2.5 cm in diameter) under room temperature. The egg masses were observed daily for hatching of yellow stem borer larvae, if any (and hatched larvae were removed), or emergence of *Trichogramma* wasps. Number of hatched larvae and emerged wasps were recorded. The emerged wasps were sorted by sex. Number of dead unparasitized eggs and dead parasitized eggs were differentiated by dissection under a stereo microscope after wasp emergence had ceased for three days. Egg mass parasitism rate was calculated by dividing numbers of parasitized egg masses with total numbers of egg masses. Egg parasitism rate was calculated by dividing numbers of parasitized eggs with total numbers of eggs.

### 2.4. Field Tests for Parasitism

Field performance of the two *Trichogramma* species was tested by releasing the wasps in paddy fields. The fields were planted to conventional rice varieties of “Deyou 8”, “Deyou 12” and “Deyou 16” that were at heading to milking stage when the experiments were conducted. Insecticides were not applied for one week before releasing of *Trichogramma* and during the experiment period.

**Figure A1:**
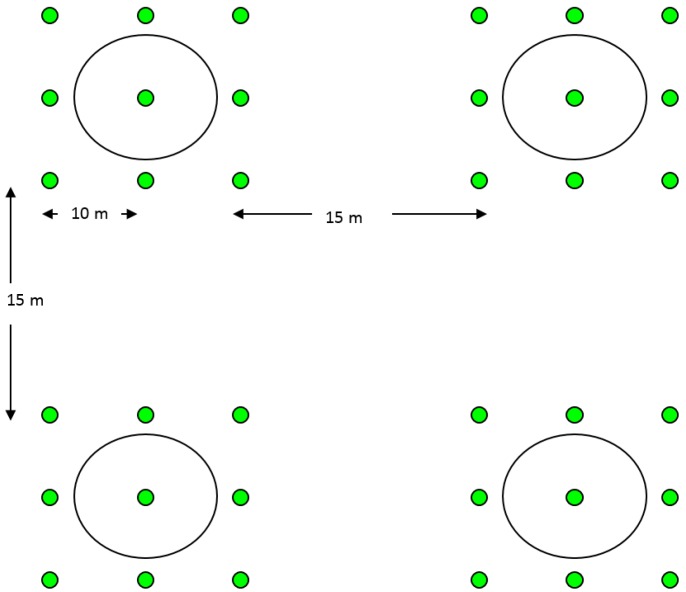
Design of field trial with *Trichogramma* wasps targeting yellow stem borer egg masses. Green circles indicate releasing points in each plot. A total of 28 plots were randomly chosen in the paddy field with at least 15 m distance from edge to edge. Two species of wasps were released in three densities of 500, 1000 and 2000 per releasing point respectively.

Both *T. japonicum* and *T. chilonis* were released at 100 points per ha at 500, 1000 and 2000 wasps per point, corresponding to release densities of 50,000, 100,000 and 200,000 wasps/ha, respectively. For each combination of release density and *Trichogramma* species, there were four replicates (field plots). Another four plots were used as control. Each plot covered an area of 900 m^2^ (30 m × 30 m), and was at least 15 m (edge to edge) away from the neighboring plots. Tricho-cards were released in a plot at nine points that were 10 m apart from each other. The plots were randomly assigned to treatments in the field (Appendix Figure A1).

On the same day when the blackened (ready-to-emerge) tricho-cards were released, sentinel YSB egg masses less than 24 h old were placed in the plots. The sentinel egg masses were obtained from the established cage rearing. For each plot, 10 YSB egg masses together with a rice leaf segment were randomly fixed on either leaf side by a stapler. Forty-eight hours after releasing, YSB egg masses were recollected from the fields and taken back to laboratory. In addition to re-collecting the actively placed egg masses, all other YSB egg masses found were also taken back to the lab. Egg masses were kept under natural conditions and observed for number of hatched larvae, emerged wasps and dead eggs, as described in the cage tests. The local weather in July and August of 2013 was characterized by small rains occurring at least once a day, with a temperature range of 22 °C to 33 °C and an average wind speed less than 3.4 m/s.

### 2.5. Morphological Observations

Morphological parameters potentially affecting the ability to parasitize YSB eggs were measured for field-collected and mass reared *Trichogramma*. The field-collected *T. japonicum* were from YSB egg masses collected in 2014 from paddy fields in Husa Township where *T. japonicum* had been released for one year. The mass reared *T. japonicum* and *T. chilonis* were produced by the local *Trichogramma* rearing facility in Mangshi, Dehong Prefecture, where the wasps had been reared for >20 generations on eggs of *C. cephalonica*. Body length, ovipositor length and hind tibia length were measured under a stereo microscope to 15 wasps for each of the field-collected *T. japonicum* and the mass reared *T. japonicum* and *T. chilonis*.

### 2.6. Data Analysis

Egg parasitism rate was calculated as: (number of dead parasitized eggs + number of emerged wasps)/(total number of eggs); egg mass parasitism rate, as number of parasitized egg masses/total number of egg masses; and emergence rate, as number of emerged wasps/(number of dead parasitized eggs + number of emerged wasps). For the field test, mean values of the parameters were calculated for a plot and then tested with a two-factorial ANOVA for the two factors “species” and “densities”. For the cage test, means of the parameters between the two species were differentiated by *t*-test or Mann-Whitney *U*-test. All proportional data were arcsin-squareroot transformed before analyses, which were performed with the program IBM SPSS Statistics 19.0.0 (SPSS Inc., Chicago, IL, USA).

## 3. Results

### 3.1. Performance of the Trichogramma Species on Substitute Host

Ninety percent of the *Sitotroga* eggs were parasitized in the test. From a total of 500 eggs exposed for each of the two *Trichogramma* species, there were 289 ± 46 *T. japonicum* wasps and 245 ± 18 *T. chilonis* wasps that emerged, respectively. No significant difference was found in mean emergence rates between *T. japonicum* (64.2% ± 10.2%) and *T. chilonis* (54.4% ± 4%; *t* = 0.902, *p* = 0.42).

### 3.2. Cage Tests

**Figure 1:**
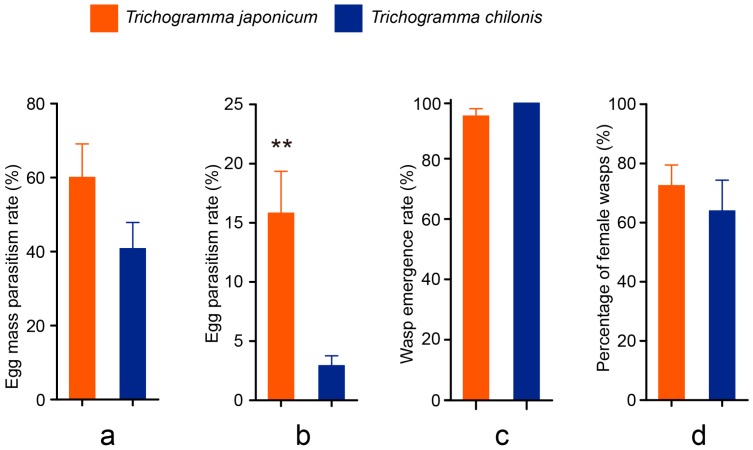
Parameters indicating performances of *T. japonicum* and *T. chilonis* in the cage test. Bar charts show comparisons of (**a**) egg mass parasitism rate; (**b**) egg parasitism rate; (**c**) emergence rate; and (**d**) proportion of females, between the two *Trichogramma* species. Error bars indicate + standard error (SE) and asterisks indicate significant difference observed at *p* < 0.01.

A total of 77 YSB egg masses were collected from the cage tests, 37 for the *T. chilonis* treatment and 40 for the *T. japonicum* treatment. Mean parasitism rates of egg masses were 60.0% ± 9.1% for *T. japonicum* and 40.7% ± 7.1% for *T. chilonis* and did not significantly differ from each other (*t*_6_ = 1.67, *p* = 0.145; Figure 1a).

In contrast, *T. japonicum* wasps showed a significantly higher parasitism rate on yellow stem borer eggs (15.8% ± 22.2%) than *T. chilonis* (2.8% ± 5.0%, *t*_6_ = 3.51, *p* = 0.0011; Figure 1b). The parasitism rate altogether was rather low for both species. There were an average of 1.73 ± 2.04 *T. japonicum* wasps and 0.65 ± 0.98 *T. chilonis* wasps emerging from an egg mass. Overall emergence rate for *T. japonicum* was 94.5%, which was not significantly different from that of *T. chilonis* (100%; Mann-Whitney *U* test, *U* = 157.5, *p* = 0.521, Figure 1c). Sex ratios (measured as proportion of females) were 71.2% for *T. japonicum* and 62.5% for *T. chilonis*, with no difference observed between the two species (*t*_6_ = 0.703, *p* = 0.486, Figure 1d).

### 3.3. Field Tests

**Figure 2:**
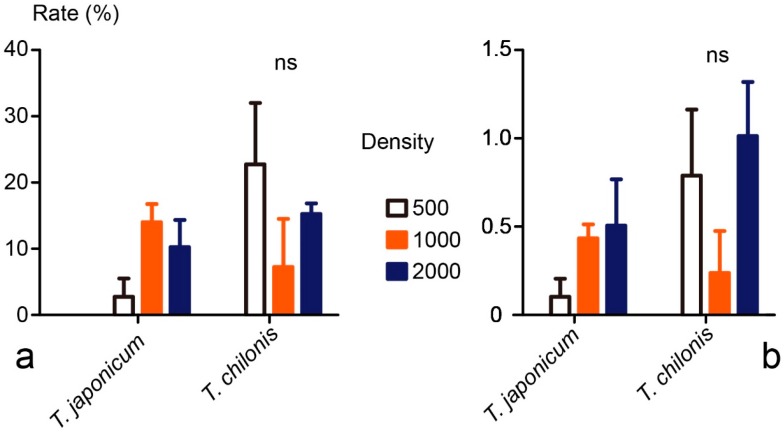
Results from field experiment to assess yellow stem borer (YSB) parasitism rates of two *Trichogramma* species at three release densities (500, 1000 or 2000 wasps per release point, *n* = 4 for each treatment). Error bars indicate + SE. (**a**) YSB egg mass parasitism rate; (**b**) YSB egg parasitism rate.

**Figure A2:**
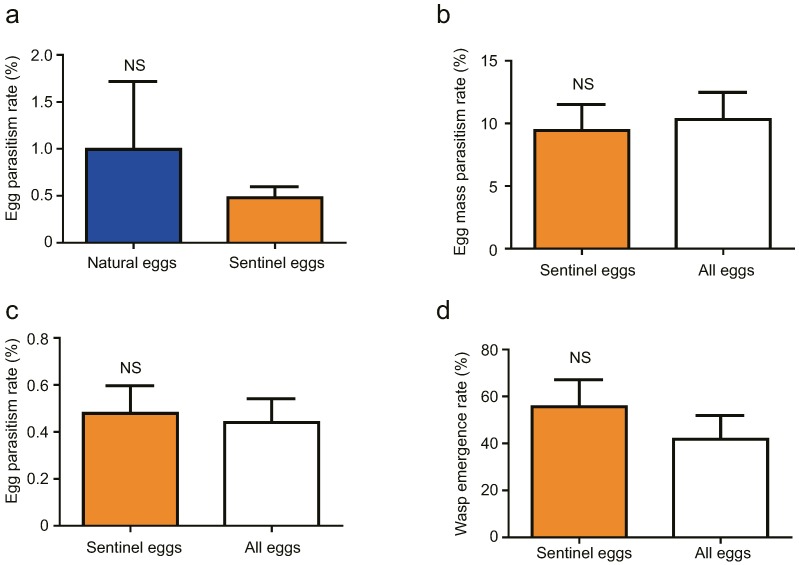
Comparison of data from sentinel and naturally laid YSB eggs. *t*-test, *n* = 28, error bars indicate + SE. (**a**) Egg parasitism rate comparison between natural and sentinel eggs. *t* = 0.67, *p* = 0.5; (**b**) Egg mass parasitism rate comparison between sentinel and all eggs. *t* = 0.29, *p* = 0.77; (**c**) Egg parasitism rate comparison between sentinel and all eggs. *t* = 0.25, *p* = 0.8; (**d**) Wasp emergence rate comparison between sentinel and all eggs. *t*_33_ = 0.9, *p* = 0.37.

**Figure A3:**
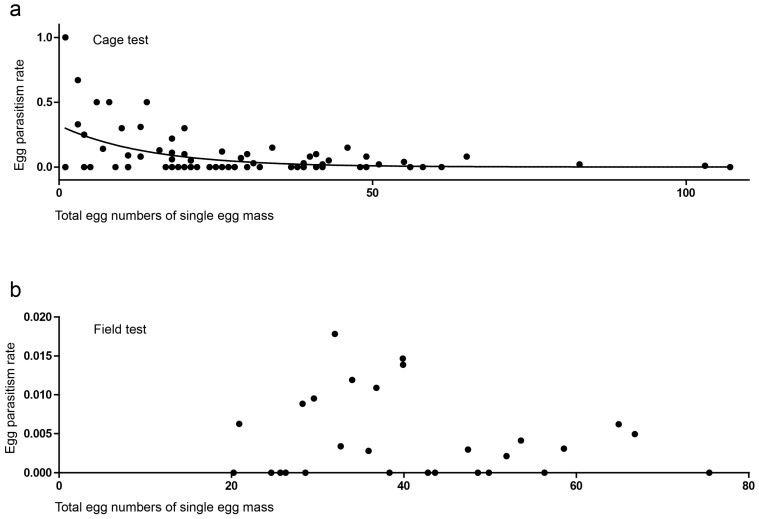
Non-linear regression between egg mass size and parasitism rate. (**a**) data from cage tests. Pearson correlation: *R*^2^ = −0.349, *p* = 0.002; (**b**) data from field tests. Pearson correlation: *R*^2^ = −0.149, *p* = 0.451.

In total, 370 YSB egg masses were recollected from the 28 plots of which 280 were sentinel egg masses and 90 were naturally laid. This included 43 egg masses from control plots, 165 from *T. japonicum* released plots and 162 from *T. chilonis* released plots. Because no significant difference was observed between sentinel eggs and naturally laid eggs in egg parasitism rates (Appendix Figure A2), data from all egg masses were pooled for further analysis. For the 43 egg masses collected from control plots, no attack by *Trichogramma* was observed, resulting in zero parasitism rate. In the *Trichogramma* release plots, parasitism rates of YSB egg masses were 9.0% ± 7.6% and 15.1% ± 14.1% for *T. japonicum* and *T. chilonis*, respectively. Rather low parasitism rates of YSB eggs were found in the present study with 0.35% ± 0.36% and 0.68% ± 0.66% for *T. japonicum* and *T. chilonis*, respectively. No significant differences were observed between the two *Trichogramma* species, neither for egg mass parasitism rate (excluding the control plots showing zero parasitism, ANOVA, *F*_1,18_ = 0.84, *p* = 0.37) or for egg parasitism rate (*F*_1,18_ = 1.20, *p* = 0.29, Figure 2). The emergence rates were 84.4% ± 19% for *T. japonicum* and 83.4% ± 15% for *T. chilonis* (*F*_1,18_ = 1.7, *p* = 0.2). Furthermore, no significant differences were found among the three release densities tested, neither for egg mass parasitism (*F*_2,18_ = 0.42; *p* = 0.66) nor for egg parasitism (*F*_2,18_ = 1.70 *p* = 0.21, Figure 2). A significant interaction effect was observed between wasp species and release density for egg mass parasitism rate (*F*_2,18_ = 4.14, *p* = 0.033) but not just for egg parasitism rate (*F*_2,18_ = 3.21, *p* = 0.064). For cage tests, a weak but significant negative correlation was found between egg mass size (indicated by total egg number of single egg mass) and egg parasitism rate (Appendix Figure A3, Pearson correlation: *R^2^* = −0.349, *p* = 0.002). Yet no significant correlation could be observed between egg mass size and egg parasitism rate in field tests (Pearson correlation: *R^2^* = −0.149, *p* = 0.451).

### 3.4. Morphological Comparison

**Figure 3:**
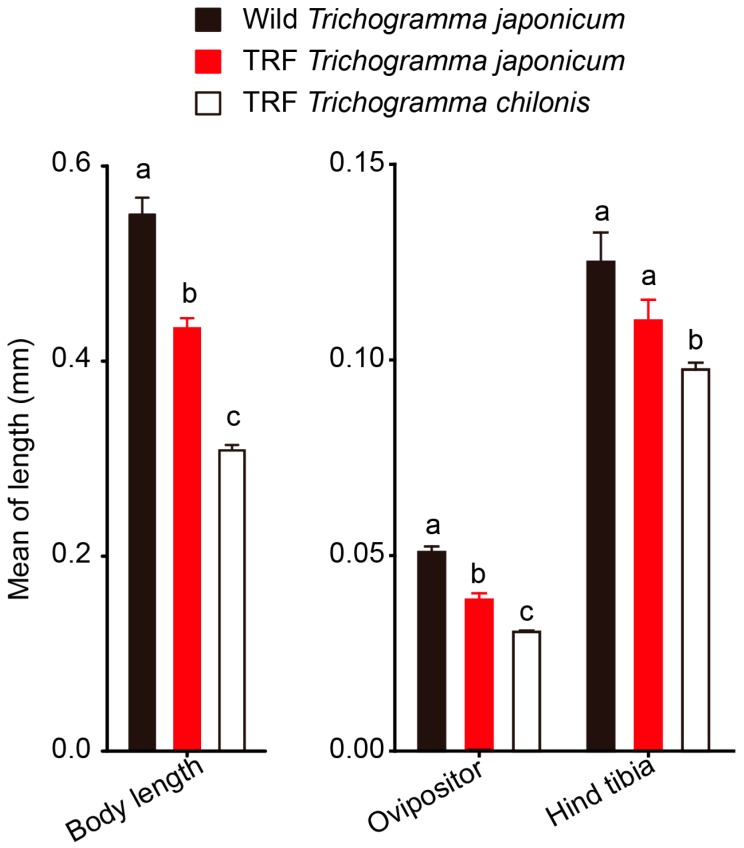
Comparison of body length, ovipositor length and hind tibia length among *Trchogramma* species/strains either collected from yellow stem borer egg masses from paddy fields (wild *T. japonicum*) or reared in *Trichogramma* rearing facilities (TRFs) on eggs of *C. cephalonica* (*n* = 15). Error bars indicate + SE. Lower case letters indicate significant differences among the three species/strains (one-way ANOVA followed by Tukey HSD multiple comparison).

**Figure A4:**
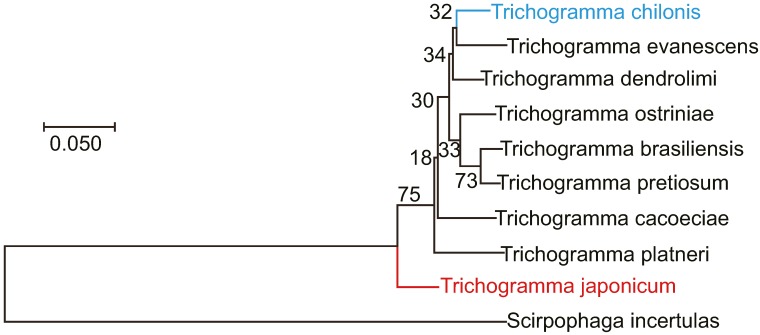
The *Trichogramma* evolutionary history was inferred using the Neighbor-Joining method [42]. The optimal tree with the sum of branch length = 0.94808551 is shown. The percentage of replicate trees in which the associated taxa clustered together in the bootstrap test (1000 replicates) are shown next to the branches [43]. Scale indicates evolutionary distances among species. A total of nine *Trichogramma* species and yellow stem borer were picked up for phylogenetic analysis. Ten cytochrome oxidase subunit I genes were introduced from gene bank for the analysis including *T. japonicum* (gi|727353589|), *T. chilonis* (gi|700682216|), *T. dendrolimi* (gi|449043448|), *T. ostriniae* (gi|74483381|), *T. brasiliensis* (gi|74483391|), *T. platneri* (gi|1052465561|), *T. pretiosum* (gi|742527366|), *T. evanescens* (gi|742527376|), *T. cacoeciae* (gi|700676774|), and *Scirpophaga incertulas* (gi|633259618|). The tree is drawn to scale, with branch lengths in the same units as those of the evolutionary distances used to infer the phylogenetic tree. The evolutionary distances were computed using the Maximum Composite Likelihood method [44] and are in the units of the number of base substitutions per site. Evolutionary analyses were conducted in MEGA7 [45].

The field-collected *Trichogramma* wasps were identified as *T. japonicum* (Appendix Figure A4). Significant differences between field-collected *T. japonicum* and mass reared *T. japonicum* or *T. chilonis* were found for body length (ANOVA, *F*_2,36_ = 93.5, *p* < 0.001), ovipositor length (*F*_2,36_ = 51.4, *p* < 0.001) and hind tibia length (*F*_2,36_ = 5.88, *p* = 0.0062, Figure 3). In particular, the field-collected *T. japonicum* were larger than the mass reared conspecifics (*Tukey* HSD test, body length: *p* < 0.001, ovipositor length: *p* < 0.001), although no significant difference was found for hind tibia length (*p* = 0.134). Furthermore, the mass reared *T. japonicum* were larger than the mass reared *T. chilonis* for two tested parameters (body length: *p* < 0.001, ovipositor length: *p* < 0.001, hind tibia length: *p* = 0.242)

## 4. Discussion

In the present study, we tested two *Trichogramma* species collected in the target region for their potential as a biological control agent of YSB, the main pest of rice in the target region. No conclusion can be drawn as to which of the two tested species might be better, since results from cage and field tests were not consistent and slightly contradictory. Overall, parasitism rates obtained for *T. japonicum* and *T. chilonis* in both the cage and field tests were relatively low and raise concerns as to whether these species could be successfully applied. However, a number of reasons may have contributed to low egg mass parasitism rates in the field. First of all, it cannot be ruled out that the quality of the released material is impaired, which may be due to being mass produced for many generations on *Sitotroga* [26,29], or due to sub-optimal conditions during transportation of the tricho-cards from the production facility in Hengshui to the release site in Husa Township. The low emergence rates found in the substitute host tests on *Sitotroga* eggs in the present study provide some evidence that quality of the wasps used in the study here was below optimum. It is furthermore well known for field release studies with inundative releases in plots that dispersal from these plots can be an issue and this has also been shown for *Trichogramma* [30]. Even though the release area was a bit larger than the area from which measurements were taken, dispersal over 10–20 m can happen for *Trichogramma* with corresponding effects on parasitism. In addition, the experiment was run over a short time only to avoid too much loss of egg masses due to predation. It is also likely that not all *Trichogramma* emerged during the first day of the experiment, i.e., fewer *Trichogramma* wasps may have been active during the length of the experiment than would be anticipated from the release rate. Last but not least, *Trichogramma* wasps may have had difficulties to find egg masses because they were placed experimentally on leaves which may quickly dry in the field and thus become unattractive. In light of the above considerations and compared to other studies on similar scales, the relatively low parasitism rates may be partly explained; rates would perhaps be higher in larger scale releases. Also, the fact that this study demonstrated no consistent effect of the release density indicates that unknown factors may have played a role in this field experiment.

However, despite the fact that egg mass parasitism may have been underestimated in the present study, a particular concern is still the low parasitism rates that were found for eggs, i.e., even though an egg mass might have been parasitized, *Trichogramma* females only parasitized a small proportion of that egg mass. This may be related to the specific features of YSB egg masses which on one hand consist of several layers of eggs, and on the other hand are also covered with hairs provided by the YSB female moth. In general, few studies have been carried out to test *Trichogramma* wasps on yellow stem borers and in particular, field surveys have mostly only reported egg mass parasitism rates instead of egg parasitism rates for *Trichogramma* wasps toward YSB. For egg masses, exceptional rates of 100% parasitism could be reached for *T. japonicum* toward the first generation of YSB eggs in the fields [11]. In contrast, several surveys have reported that parasitism rates of YSB eggs by *Trichogramma* in paddy fields ranged only from 2.1% to 23.1% [31,32,33]. These rates are considerably lower than those generally found for striped stem borer eggs, where parasitism rates reach up to 85.8% [6,21].

Hairs of pests have been proved to be related to both direct [34] and indirect [35] protection from natural enemy attacks. Similarly, due to the special hair cover structure and layers of eggs inside, YSB eggs usually suffer less from egg parasitoid wasps [25]. This is supported by Lou et al. [11] who recently showed that the eggs from deeper layers of YSB egg masses usually escape from being parasitized by *Trichogramma* wasps. At the high densities used for the field cage study here (100 wasps per 10 egg masses), it may be concluded that wasps would parasitize all eggs they could possibly reach which would suggest about 21% for *T. japonicum* and 4% for *T. chilonis* on small egg masses and 4.7% for *T. japonicum* and 1.9% for *T. chilonis* on larger egg masses. In fact, a negative correlation of egg parasitism rates with egg mass size was observed in cage tests, which indicates that small egg masses were more likely to be parasitized in a higher proportion than large egg masses. No such correlation was found in the field test. We were not able in the present study to precisely analyse the rate of parasitization of eggs in the different layers of egg masses, and additional studies would be worthwhile to deepen our understanding on the factors underlying our findings. Even though the hairs on the egg surface are likely impairing parasitism rates, *Trichogramma* offspring may be expected to benefit from hairs of parasitized YSB egg masses [36] as predation risk is reduced compared to *Trichogramma* offspring inside striped stem borer or rice leaf folder eggs [34,35] thus increasing offspring survival rate [37].

In the experimenta, no *Trichogramma* at all were found in the control fields or during additional collections in Husa Town [38]. Although the reasons for this remain unknown, it may be speculated that the high rate of recent pesticide applications is a contributing factor. Interestingly, *Trichogramma* wasps were recovered two months after the last of six releases that were conducted in 20 hectares of demonstration paddy fields located in Mangshi, Dehong, Yunnan Province, indicating that a natural *T. japonicum* population has successfully established there. It is known that body size of host parasitoid wasps can be determined by host egg sizes [39,40], and we found that field–collected *T. japonicum* strains were significantly larger than lab strains, produced on rather small eggs of the factitious host *Sitotroga*, and also had longer ovipositors. Larger body sizes of parasitoids are generally related to higher egg load, lifetime fecundity, ability to disperse, host locating ability, and thus may contribute to increased parasitism rates [39,40,41]. This suggests that *Trichogramma* wasps reared in small eggs might encounter difficulties when facing larger eggs or packs of egg masses, especially when they are covered with hairs in the field. In this case, second generation wasps emerging from field hosts may be more successful in parasitizing YSB eggs.

## 5. Conclusions

In conclusion, the overall low parasitism rates found in the present study suggest that the two *Trichogramma* species tested would not be highly successful for inundative biological control of YSB, in particular because of the low attack of eggs observed. However, considering the limitations of such an experimental study (see points discussed above) and likely higher success rates of larger wasps emerging from the field host, application of *Trichogramma* could have a positive effect on YSB pest control based on inundative or even inoculative releases in the longer term, and further studies will be needed to fully understand the potential, but also constraints, of the present system.

## Appendix A

**Table A1:** Quality parameters of *Trichogramma* cards provided by TBCC *.

Species	Area (cm^2^)	Number of Cards Produced Per mL eggs	Total Egg Number per Card	Parasitized Egg Number Per Card	Emerged Wasp Number Per Card	Parasitism Rate (%)	Emergence Rate (%)	Female Rate (%)
*T. japonicum*	1.8	23	1087	623.9	547	57.4 + 1.1	87.8 + 1.2	75.7 + 1.0
*T. chilonis*	1.8	23	1087	545.8	506.2	50.2 + 0.7	92.8 + 0.9	74.2 + 0.8

* Tianyi Biological Control Company Inc., Hengshui, Hebei Province, China.
